# Tranexamic acid in primary total knee arthroplasty without tourniquet: a randomized, controlled trial of oral versus intravenous versus topical administration

**DOI:** 10.1038/s41598-018-31791-x

**Published:** 2018-09-11

**Authors:** Duan Wang, Hao-Yang Wang, Chang Cao, Ling-Li Li, Wei-Kun Meng, Fu-Xing Pei, De-Hua Li, Zong-Ke Zhou, Wei-Nan Zeng

**Affiliations:** 1 Department of Orthopedics, West China Hospital/West China School of Medicine, Sichuan University, Chengdu, 610041 P.R. China; 2 Department of Cosmetic Plastic and Burns surgery, West China Hospital, Sichuan University, Chengdu, 610041 P.R. China; 3 Key laboratory of Obstetric and Gynecologic and Pediatric Disease and Birth Defects (Ministry of Education), West China Second University Hospital, Sichuan University, Chengdu, 610041 P.R. China; 4 Quality Assessment Office, West China Second University Hospital, Sichuan University, Chengdu, 610041 P.R. China; 5 Center for Joint Surgery, Southwest Hospital, Third Military Medical University, 30# Gaotanyan Street, Chongqing, 400038 P.R. China

## Abstract

Abundant literature confirms that intravenous (IV) and intra-articular (IA) administration of tranexamic acid (TXA) reduces blood loss in total knee arthroplasty (TKA). Oral formulations of TXA exhibit profound cost-saving benefits. However, comparisons of the clinical efficacy among three different modalities of TXA administration have not been previously investigated in the setting of TKA with no closed suction drain and tourniquet. A total of 180 patients undergoing TKA were randomized to receive 2-g oral TXA 2 hours preoperatively, 20-mg/kg IV TXA 5 minutes prior to incision, or 2-g IA TXA. The primary outcome was 72-hour blood loss. Secondary outcomes were reductions in hemoglobin, the rate of transfusions, and adverse events. No significant differences were identified with regard to **t**he mean 72-hour blood loss among the three groups (1003 mL in oral group, 1108 mL in IV group, and 1059 mL in IA group, respectively). Similarly, hemoglobin reduction was equivalent among the groups. Only one patient in IV group exhibited deep venous thrombosis. No difference was identified regarding transfusion rates. Oral TXA results in similar blood loss in TKA, with a profound cost-saving benefit, compared with the IA and IV formulations.

## Introduction

Total knee arthroplasty (TKA) is associated with substantial intra- and postoperative blood loss that may carry a substantial risk of anemia and allogeneic transfusions^1,2^. Allogeneic blood transfusion may lead to adverse outcomes (i.e., infection and myocardial infarction), which increases morbidity, mortality, and cost^3,4^. Various blood-saving protocols, including blood-salvaging techniques, autologous blood transfusion, and cryotherapy, as well as the perioperative administration of antifibrinolytic agents such as tranexamic acid, were adopted to reduce bleeding and allogeneic blood transfusions with varied success^5,6^.

Tranexamic acid (TXA) is a synthetic derivative of the amino acid lysine that prevents plasminogen activation via blockade of the lysine binding site of plasminogen, which promotes the coagulation process^7^. Numerous randomized controlled trials confirmed that perioperative intravenous (IV), intra-articular (IA), and oral routes of TXA administration exhibited beneficial blood-saving effects and reduced transfusion requirements without apparently increasing the risk of postoperative complications in comparison with placebo^8–10^. Most studies focused on the investigation of IA and IV modalities of TXA administration, but only two prospective studies investigated the efficacy of oral TXA administration compared to IV or IA TXA in TKA when this trial was designed^11,12^. However, the 1st study^12^ involved two groups (oral vs. IV), and the 2nd study^11^ involved four groups, including the use of a postoperative drain and a tourniquet, which enhances local fibrinolysis and affects bleeding kinetics^13^. No tourniquet or postoperative drain was used in our center on the basis of a well-established fast-track setup focusing on multimodal analgesia strategy^14^, perioperative blood-saving management^15,16^, and early function recovery^17^. Compared with an equivalent IV formulation, the oral TXA dosage produces a cost savings of $33 to $94 per dose, leading to a cost difference of about 70.2% to 90.4%^18^. However, the blood-sparing efficacy of oral TXA remains unclear in TKA without tourniquet and postoperative closed suction drain.

Therefore, our randomized controlled trial was performed with an enhanced-recovery protocol to compare the efficacy of oral administration of TXA with that of IA and IV administration of TXA without the use of closed suction drain and tourniquet. We hypothesized that oral administration of TXA would produce an equivalent reduction in postoperative blood loss compared with IA and IV administration of TXA, with a better cost-effectiveness profile.

## Materials and Methods

### Study design and Participants

This single-center, prospective, randomized, double-blinded study was performed in the Department of Orthopaedic Surgery at West China Hospital. Following the approval of the Clinical Trials and Biomedical Ethics Committee of Sichuan University (No. 201302008), the study was registered in the Chinese Clinical Trial Registry (ChiCTR-INR-17010965). Written informed consent was obtained from all participants. All the methods were conducted according to the CONSORT statement^19^.

All adult patients (≥18 years of age) with primary knee osteoarthritis who were scheduled for elective primary unilateral total knee replacement from March 2016 to January 2017 were assessed for eligibility. At our center, the perioperative managements were performed according to a well-established fact-track protocol. The criteria for exclusion included secondary osteoarthritis (e.g., post-septic arthritis and post-traumatic arthritis), simultaneous bilateral or revision TKA, allergic reaction to TXA, history of major comorbidities (severe arterial thromboembolic event, severe renal failure, or severe pulmonary disease), history of hematopoietic disease, history of pulmonary embolism (PE) or deep venous thrombosis (DVT), alcohol or drug abuse, and current anticoagulant therapy (warfarin or heparin) within one week.

### Randomization and trial intervention

The recruited patients were randomized to one of three interventions. The oral group received a total dose of 2 g of oral TXA with use of four 500-mg tablets approximately 2 hours before incision. The patients in the oral group were also administered 100 mL of an IV and IA placebo solution (normal saline solution) in a manner identical to administration in IV and IA groups, respectively. Oral TXA dosing and timing were based on pharmacokinetic studies, which demonstrate that 2.0 grams of oral TXA reaches peak levels after about 2 to 3 hours and falls below the therapeutic threshold 6 hours after administration^10^. The IV group received a 20-mg/kg dose of TXA in 100 mL of normal saline solution administered intravenously 5 minutes prior to incision and 100 mL of a placebo solution administered intra-articularly, based on previous efficacy studies^20^. The IA group received a 2-g dose of TXA, diluted in 100 mL of saline solution administered intra-articularly according to previous studies that demonstrated the efficacy of TXA in reducing bleeding at doses of 1.5 g to 3 g^21,22^, and a placebo dose of 100-mL saline solution administered intravenously. IA TXA was administered at two time points: (1) the open joint surface was soaked with 50 mL of a 1-g TXA solution following component implantation and was left in contact with the tissue for five minutes; (2) the remaining 50 mL of a 1-g TXA solution was given using a needle to penetrate the tissue of knee capsule before capsule closure^16,23^. The oral and IV groups were also subjected to the 2-stage exposure to TXA placebo as described for the IA group. The patients in the IV and IA groups also received a total dose of 2-g small placebo pills that were identical in appearance but contained no active ingredient.

A random allocation sequence was computer-generated online (www.randomization.org), with an allocation ratio of 1:1:1 and a block size of 60. One of two surgeons responsible for all arthroplasties enrolled patients, and all the patients were screened in clinics. Dedicated research personnel rechecked the inclusion and exclusion criteria of the patients. The patients were assigned a unique randomization number, and patient assignments were concealed into consecutively numbered opaque-sealed envelopes. An envelope was opened on the morning of operation, and a research pharmacist (who was not otherwise involved with the collection of patient data or patient care) handled all the study drug preparations to ensure identical appearance and blinding. This information was linked to a confidential database, which was used for collection and analysis by an independent research statistician. The patients, surgeons, anesthesiologists, data collectors, research assistants, and outcome assessors were blinded.

### Surgical technique and postoperative management

The operations were performed by two senior surgeons. In all patients, standardized general anesthesia and a medial para-patellar approach were used. Perioperative multimodal analgesia was standardized and consisted of an adductor canal block and periarticular multi-site infiltration^14^ as well as standard analgesia^24^. The tourniquet and intra-articular closed suction drain were not used^17,25^.

Venous thromboembolism prophylaxis was administered as standard practice, with a combination of mechanical and chemical thromboprophylaxis. A portable intermittent inflatable calf pump was used for mechanical prophylaxis, and lower-extremity strength training was conducted on the day after surgery. While hospitalized, chemical prophylaxis consisted of subcutaneous administration of low-molecular-weight heparin (2000 IU) beginning 8 hours postoperatively, which was then administered once daily (4000 IU). Rivaroxaban (10 mg orally, Xarelto; Bayer, Leverkusen, Germany) was administered daily, which continued for 10 days after discharge.

During hospitalization, a standardized blood-transfusion protocol was followed for all patients on the basis of the perioperative transfusion guidelines of the Chinese Ministry of Health. Red blood cells were transfused when hemoglobin (Hb) levels were <70 g/L in asymptomatic patients or when a patient exhibited any organ dysfunction associated with anemia, regardless of Hb concentration.

### Outcome measures

The primary outcome measure was 72-hour blood loss. The calculated blood loss was estimated via application of the Gross formula^26^, and total blood value was calculated using the formula described by Nadler *et al*.^27^: Patient blood volume (PBV) = k_1_ x height (m) + k_2_ x weight (kg) + k_3_ (k_1_ = 0:3669, k_2_ = 0:03219, and k_3_ = 0:6041 for men; k_1_ = 0:3561, k_2_ = 0:03308, and k_3_ = 0:1833 for women). Estimated total blood loss = PBV x (Hct_pre_ − Hct_post_)/Hct_ave_ (Hct_pre_ = the initial preoperative hematocrit level. Hct_post_ = the postoperative 72-hour hematocrit level during hospitalization. Hct_ave_ = the average of the Hct_pre_ and Hct_post_). The total blood loss was equivalent to the volume transfused plus the blood loss when an allogenic transfusion was performed^28,29^.

The secondary outcome measures were Hb and hematocrit (Hct) level, Hb drops, coagulation indicators on postoperative days (PODs) 1 and 3, the amount of IV fluid, and intraoperative blood loss. Other secondary outcome measures were thromboembolic complications, the rate of blood transfusions, the total number of blood units transfused, adverse events, the costs of allogeneic blood transfusion and TXA, 30-day mortality, 90-day readmission, hospital stay, and knee function.

Intraoperative blood loss was evaluated via measurement of suction drain contents and surgical swabs^30^. All the patients received examination for clinical symptoms of DVT every day during hospitalization. Both lower limbs were examined using a diagnostic Doppler ultrasound on postoperative day 3, or earlier, if a patient exhibited any clinical symptom (e.g., limb swelling or calf pain). All the adverse events that occurred during the first 90 days were recorded postoperatively.

### Sample size

Sample size requirement was determined on differences in the calculated 72-hour blood loss. A review of a historical cohort of 114 patients undergoing primary TKA using a similar fast-track setup at our center from September 2014 to June 2015 revealed that the 72-hour total blood loss was 1104 mL, with a standard deviation of 254 mL. We assumed a clinically important blood loss difference of 110 mL, which was equivalent to a reduction in blood loss of 10% in the historical cohort. The sample size was calculated using a fixed-effect one-way analysis of variance, with an assumed alpha level of 5% and a power of 90%. A minimum of 147 patients was required. Sixty patients per arm were needed to compensate for a 20% expected dropout rate.

### Statistical analysis

The distributions of all the variables were tested using the Kolmogorov-Smirnov test prior to analyses. Distributions of the demographic and perioperative data were evaluated with summary statistics, including means and standard deviations (normalized data) or medians and interquartile ranges (non-normalized data) for quantitative data as well as numbers and percentages for qualitative data. One-way analysis of variance (ANOVA) with Tukey’s post hoc test was used for normally distributed continuous variables, and Kruskal-Wallis with Nemenyi post hoc test was used for skewed continuous variables. The categorical variables were analyzed with use of Chi-square test or Fisher’s exact test. All data were analyzed using SPSS software (Version 21.0; SPSS Inc., Chicago, IL). A P <0.05 (two-sided) was considered significant. The statistical power of the study was calculated using G-Power software (Version 3.1.9.2, Germany).

## Results

### Patients

**Figure 1:**
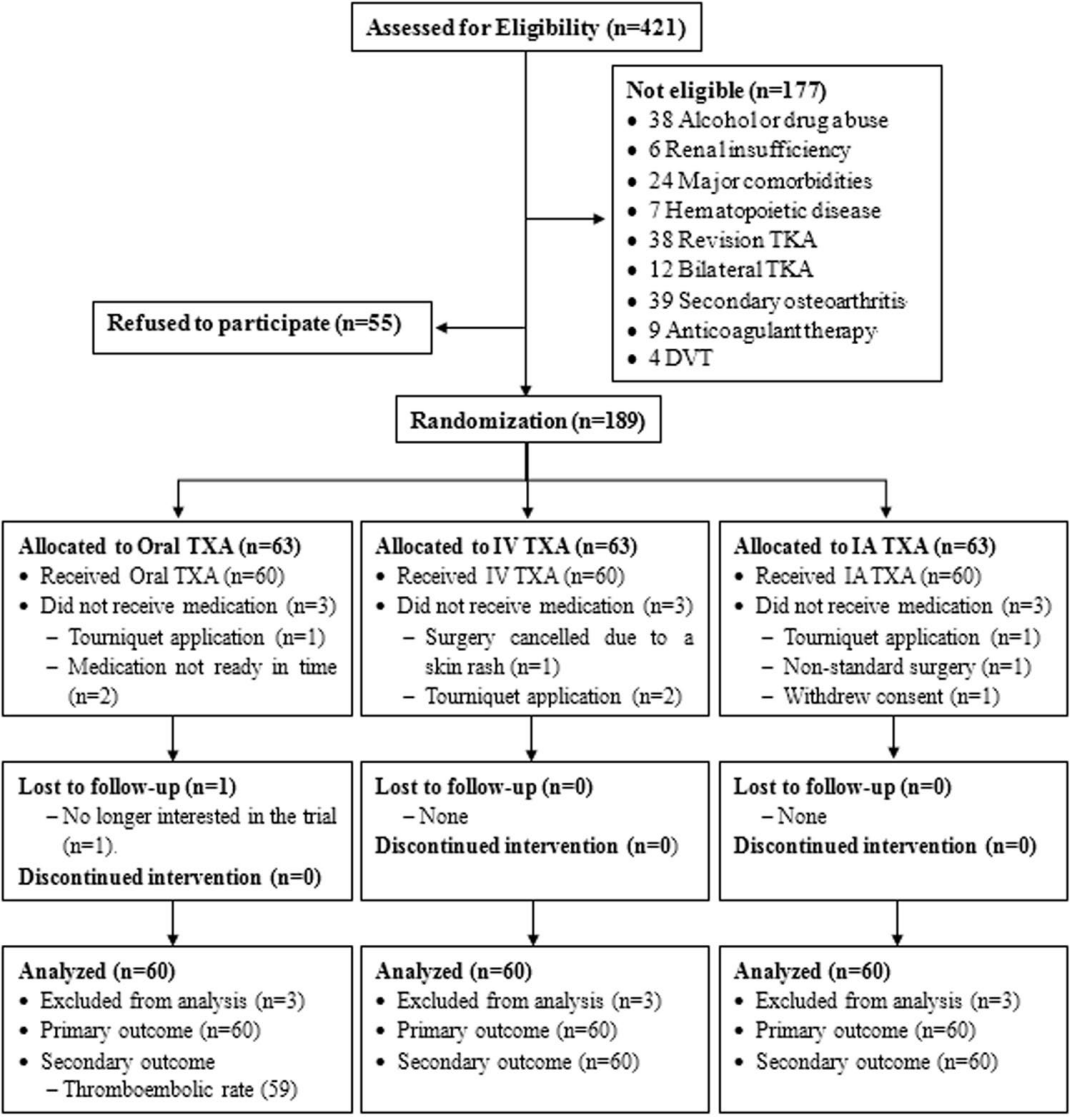
CONSORT (Consolidated Standards of Reporting Trials) flow diagram.

A total of 421 patients who were scheduled for TKA were screened for eligibility from March 2016 to January 2017, and a total of 241 patients were excluded (Fig. 1). Therefore, 180 enrolled study participants were included in the randomization in terms of interventions (oral TXA, IV TXA, or IA TXA; 60 per arm). One patient in the oral group was lost to follow-up for the analysis of thromboembolic events due to lack of interest in this trial. However, this patient was included in the primary outcome analysis. The baseline characteristics were similar between groups (Table 1).

**Table 1:** Preoperative characteristics.

Variable	Oral TXA Group (N = 60)	IV TXA Group (N = 60)	IA TXA Group (N = 60)	P value^¶^
Demographic characteristics				
Age^†^ (yr)	63.91 ± 13.07	66.90 ± 9.48	63.20 ± 11.75	0.19
Male sex^‡^ (no. [%])	18 (30%)	15 (25%)	16 (27%)	0.82
Height^†^ (cm)	1.58 ± 0.69	1.57 ± 0.67	1.57 ± 0.57	0.61
Weight^†^ (kg)	63.21 ± 11.17	61.87 ± 9.34	63.05 ± 9.75	0.73
BMI^†^ (kg/m^2^)	25.27 ± 4.17	25.04 ± 3.41	25.53 ± 3.81	0.77
Operated side, left/right^‡^ (no. [%])	39 (65%)	34 (57%)	37 (62%)	0.64
ASA classification^‡^ (no. of patients)				
I	7 (12%)	8 (13%)	7 (12%)	0.97
II	39 (65%)	40 (67%)	41 (68%)	
III	14 (23%)	12 (20%)	11 (18%)	
IV	0 (0%)	0 (0%)	0 (0%)	
Caprini score^†^ (point)	8.2 ± 0.95	8.33 ± 1.09	8.46 ± 1.19	0.41
Preop. values (blood routine)				
Hemoglobin^†^ (g/dL)	13.40 ± 1.40	13.35 ± 1.17	13.37 ± 1.23	0.98
Hematocrit^†^ (L/L)	0.41 ± 0.04	0.41 ± 0.03	0.40 ± 0.03	0.09
Platelet count^†^ (×10^9^/L)	185.28 ± 54.68	184.70 ± 51.38	188.91 ± 57.34	0.91
Red blood cell count^†^ (×10^12^/L)	4.48 ± 0.46	4.41 ± 0.40	4.52 ± 0.45	0.38
Preop. values (blood coagulation)				
Prothrombin time^†^ (s)	11.39 ± 0.65	11.29 ± 0.92	11.57 ± 0.84	0.17
INR^†^	0.98 ± 0.53	1.06 ± 0.47	1.01 ± 0.22	0.39
APTT^†^ (s)	27.24 ± 3.68	27.92 ± 2.86	28.24 ± 3.81	0.28
Fibrinogen^†^ (g/L)	2.97 ± 0.95	2.78 ± 0.92	2.57 ± 0.79	0.06
D-Dimer^†^ (mg/L)	0.91 ± 1.17	1.07 ± 1.32	0.86 ± 0.87	0.54
FDP^†^ (mg/L)	2.72 ± 2.25	3.07 ± 3.37	2.55 ± 2.19	0.55
Preop. knee function				
ROM^†^	93.10 ± 17.41	92.48 ± 15.54	94.12 ± 16.88	0.86
KSS^†^	47.38 ± 9.81	46.32 ± 11.80	47.05 ± 10.02	0.85

ASA = American Society of Anesthesiologists, INR = international normalized ratio, APTT = activated partial thromboplastin time, FDP = fibrinogen degradation product, ROM = range of motion, and KSS = knee society score. ^†^The values are presented as the mean and the standard deviation. ^‡^The values are given as the number of patient and the percentage of the group. ^¶^The p value represents the result of one-way analysis of variance for independent means for continuous variables or the chi-square test for independent proportions that included the three groups.

### Primary Outcome

The 72-hour calculated blood loss was 1003.99 ± 414.44 mL in the oral group, 1108.31 ± 392.11 mL in the IV group, and 1059.37 ± 422.99 mL in the IA group (*p* = 0.29) (Table 2).

**Table 2:** Perioperative outcomes regarding blood loss.

Variable	Oral TXA Group	IV TXA Group	IA TXA Group	P value
Total blood loss^†^ (mL)				
24 hr	593.59 ± 299.90	656.37 ± 216.30	589.89 ± 292.52	0.54
72 hr	1003.99 ± 414.44	1108.31 ± 392.11	1059.37 ± 422.99	0.29
Hemoglobin^†^ (g/dL)				
24 hr	11.5 ± 1.40	11.45 ± 1.09	11.63 ± 1.19	0.71
72 hr	10.48 ± 1.41	10.22 ± 1.11	10.38 ± 1.13	0.50
Reduction of hemoglobin^†^ (g/dL)				
24 hr	1.89 ± 1.06	1.90 ± 0.80	1.74 ± 0.93	0.58
72 hr	2.91 ± 1.13	3.13 ± 0.89	2.99 ± 1.03	0.52
Hematocrit^†^ (L/L)				
24 hr	0.35 ± 0.04	0.35 ± 0.03	0.34 ± 0.03	0.18
72 hr	0.32 ± 0.04	0.31 ± 0.03	0.30 ± 0.03	0.07
Postop. laboratory values at 72 hr				
Platelet count^†^ (×10^9^/L)	157.63 ± 48.66	156.15 ± 40.33	161.53 ± 52.25	0.86
Red blood cell count^†^ (×10^12^/L)	3.52 ± 0.43	3.41 ± 0.40	3.47 ± 0.45	0.42
Intraoperative blood loss^†^ (mL)	147.12 ± 25.64	148.92 ± 31.43	150.16 ± 28.22	0.84
Postop. IV fluid amount^†^ (mL)	2715.17 ± 375.76	2803.67 ± 351.18	2836.17 ± 434.15	0.21

^†^The values are presented as the mean and the standard deviation.

### Secondary Outcomes

No differences among groups were observed with respect to intraoperative blood loss (*p* = 0.84) or 24-hour calculated blood loss (*p* = 0.54). The mean reductions in Hb at 24 and 72 hours were 1.89 g/dL and 2.91 g/dL in the oral group, 1.74 g/dL and 2.99 g/dL in the IA group, and 1.90 g/dL and 3.13 g/dL in the IV group, with no significant intergroup differences (Table 2). There were no significant differences in terms of blood coagulation values (Table 3).

**Table 3:** Postoperative outcomes regarding blood coagulation values.

Variable	Oral TXA Group	IV TXA Group	IA TXA Group	P value
Prothrombin time^†^ (s)				
24 hr	12.13 ± 1.70	12.20 ± 1.75	11.85 ± 0.98	0.41
72 hr	11.55 ± 1.29	11.15 ± 1.34	11.44 ± 1.18	0.21
INR^†^				
24 hr	1.03 ± 0.32	1.09 ± 0.66	1.08 ± 0.64	0.82
72 hr	0.98 ± 0.65	1.02 ± 0.31	1.01 ± 0.71	0.9
APTT^†^ (s)				
24 hr	31.61 ± 5.14	32.96 ± 4.94	31.01 ± 3.72	0.07
72 hr	30.61 ± 5.41	31.35 ± 5.05	31.90 ± 3.49	0.33
Fibrinogen^†^ (g/L)				
24 hr	3.38 ± 1.35	3.36 ± 1.44	3.71 ± 1.41	0.31
72 hr	4.42 ± 1.15	4.40 ± 1.22	4.21 ± 1.17	0.55
D-Dimer^†^ (mg/L)				
24 hr	6.44 ± 7.77	7.02 ± 5.49	7.61 ± 5.41	0.59
72 hr	3.15 ± 2.05	2.77 ± 1.90	2.50 ± 1.93	0.19
FDP^†^ (mg/L)				
24 hr	22.52 ± 18.90	21.81 ± 18.55	24.24 ± 18.81	0.77
72 hr	9.29 ± 6.83	8.28 ± 8.41	8.75 ± 6.84	0.76

^†^The values are presented as the mean and the standard deviation.

Knee function was similar among the groups 3 months postoperatively. Eight patients (4.4%) were given allogeneic blood transfusion (two patients in oral group, four patients in IV group, and two patients in IA group) (Table 4). The total number of transfused units were similar among the groups.

**Table 4:** Postoperative data including adverse events.

Variable	Oral TXA Group	IV TXA Group	IA TXA Group	P value^¶^
No. (%) of patients transfused^‡^	2 (3.3%)	4 (6.7%)	2 (3.3%)	0.44
No. of units transfused (U)	4	5	5	0.96
Total transfusion cost (¥)	2750	3100	2990	0.98
Total TXA cost (¥)	480^§,#^	3341^#^	3540	<0.001
TXA cost per patient (¥)	8^§,#^	36.3^#^	29	<0.001
Length of hospital stay^†^ (d)	3 (3–6)	4 (3–7)	3 (3–6)	0.29
Operative time^†^ (min)	66.63 ± 10.96	67.56 ± 12.49	67.80 ± 12.91	0.86
Postop. complications^‡^				
DVT	0	1	0	0.37
PE	0	0	0	—
Superficial infection	1	0	1	0.6
Hematoma	0	1	1	0.6
Wound secretion	1	0	1	0.6
Gastric hemorrhage	0	0	0	—
Postop. knee function at three months				
ROM^†^	113.33 ± 8.98	115.96 ± 9.54	115.37 ± 8.33	0.24
KSS^†^	83.20 ± 2.71	84.78 ± 10.75	84.27 ± 7.56	0.52
All cause 30-day mortality^‡^	0	0	0	—
All cause 90-day readmission^‡^	0	0	0	—

DVT, deep vein thrombosis; PE, pulmonary embolism. ^†^The values are presented as the mean and the standard deviation. ^‡^The values are given as the number of patient. ^§^Significantly different from the IV TXA group. ^#^Significantly different from the IA TXA group.

The cost analysis was based on blood transfusion and TXA costs. The oral, IV, and IA TXA costs were approximately ¥ 8, ¥ 36.3, and ¥ 29 at our institution, respectively (Table 4). The lowest total TXA cost was recorded in the oral group (¥ 480) compared to the IV group (¥ 3341) and the IA group (¥ 3540) (p < 0.001). Oral TXA indicated a net savings of ¥ 2861 and ¥ 3060 per 60 treated patients when compared with the IV and IA TXA groups, respectively. However, no significant differences were identified for transfusion costs per 60 TKA patients (Table 4).

One patient in the IV TXA group exhibited DVT, which was confirmed using Doppler ultrasonography, without any clinical symptoms and was managed using the standard thromboembolism-prophylaxis protocol^31^. No PE occurred in any of the groups. The 3 groups exhibited similar numbers of other adverse events, which were all successfully resolved (Table 4).

## Discussion

Numerous studies have reported that IV and IA TXA administration represented an effective strategy to reduce postoperative blood loss and transfusions with no increased risk of thromboembolic complications in TKA^8,9,16,28^. Oral TXA formulations largely reduced costs and exhibited blood-sparing efficacy^32,33^, but only two studies investigated the efficacy of oral versus IV or IA TXA administration in total knee replacement when the trial was designed and developed^11,12^. Nevertheless, the application of closed suction drain and tourniquet was standard in these previous studies. The present study is novel because it assessed the effect of three different modalities of TXA administration in a fast-track protocol for TKA with no tourniquet or postoperative drain at the same time. The primary finding of our study was that no significant difference was identified in the blood-saving efficacy of the three different modalities, and oral TXA provided greater cost-saving benefits.

Many randomized controlled trials confirmed the safety and efficacy of IA and IV TXA administration compared to placebo during TKA, and various dosages and modalities of TXA administration exist^16,20,34^. A well-performed randomized controlled trial confirmed significantly decreased blood loss and transfusion requirements in patients treated with a single 2-g dose of IA TXA compared to the placebo^35^. A meta-analysis by Shin *et al*. demonstrated that 2-g IA TXA was a safer alternative within the therapeutic range for high-risk patients^21^. The routine dosing for TXA as an IV bolus is 20 mg/kg at our center, which exhibits therapeutic levels for 3 hours. Its efficacy and safety were confirmed in our randomized controlled trials^20,36^. Therefore, we compared 2-g oral TXA with 2-g IA and 20-mg/kg IV TXA to investigate the clinical efficacy of reducing blood loss.

Alipour *et al*.^37^ demonstrated that 1-g oral TXA administered 2 h prior to surgery exhibited blood-saving effects compared to placebo. Lee *et al*.^32^ reported that the total blood loss in patients receiving oral TXA was much smaller than that of placebo. However, only 2 trials investigated the efficacy of oral TXA compared to other routes of TXA administration in TKA^11,12^ (Table 5). Fillingham *et al*.^37^ reported that a single 2-g oral dose of TXA provided an equivalent reduction in blood loss and a lower TXA-dosage cost compared to IV TXA in a small trial. Yuan *et al*.^11^ included four study arms (oral, IV, IA, and placebo) and used drain output to roughly measure blood loss. TXA administration via any of the 3 routes significantly reduced blood loss and transfusion rates, and oral TXA similarly reduced blood loss compared to IV and IA TXA. However, a tourniquet and postoperative closed suction drain were used routinely. Moreover, the Hct in drain output gradually decreased, which may affect the accuracy of postoperative blood loss measurements^38^. Our randomized controlled trail^25^ and meta-analysis^39^ reported that the use of a drain could not significantly reduce postoperative blood loss and improve knee function following TKA. Therefore, postoperative drains are not used routinely at our center. Numerous studies indicated that tourniquet application caused muscle damage and delayed recovery^17^, increased thromboembolic event rates, and stimulated fibrinolysis with increased bleeding^40^. Moreover, some trials reported that the absence of tourniquet did not appear to affect tibial cementation penetration^41^ or fixation^42^. Therefore, the use of a tourniquet is not standard in our institution. Our study estimated changes in blood loss with adequate power. Our findings suggested that no significant differences were observed in calculated blood loss among the groups. This finding is comparable to the findings of a recent meta-analysis that demonstrated that IA TXA provided equivalent reductions in blood loss when compared with IV administration^43^. Moreover, a similar trial that was conducted later, based on this current study results, has recently been published (during the review process), confirming that oral TXA is effective even against higher IA doses^44^. However, IA or IV TXA administration should only be applied for patients with swallowing difficulties.

**Table 5:** Previously reported results of oral administration of TXA in total knee arthroplasty.

Authors	Year	Study design	Dosing regimens	Tourniquet	Drain	No. of patients		Reduced blood loss	Reduction of Hb	Thromboembolic complications		No. of transfusion	
						TXA	Control			TXA	Control	TXA	Control
Zohar *et al*.	2004	RCT	1 g TXA 60 min before surgery; 1 g TXA every 6 h for 3 times	Yes	Yes	20	20	Significant	NA	0	0	4	12
Charoencholvanich *et al*.	2011	RCT	10 mg/kg before deflation; 0.5 g oral TXA for 5 days	Yes	Yes	50	50	Significant	Significant	0	0	28	45
Alipour *et al*.	2013	RCT	1 g oral TXA before surgery; 1 g oral TXA every 6 h for 18 h postoperatively	Yes	Yes	26	27	Significant	NA	0	0	NA	NA
Lee *et al*.	2017	RCT	1 g oral TXA 2 hours before surgery; 1 g oral TXA 6 and 12 h postoperatively	Yes	Yes	95	95	Significant	Significant	1 DVT/1 PE	1 DVT/0 PE	1	3
Yuan *et al*.	2017	RCT	20 mg/kg oral TXA 2 hours before surgery; 2 g oral TXA 12 h postoperatively	Yes	Yes	140	140	NA	Significant	1 DVT/0 PE	1 DVT/0 PE	15	36

NA = not available, RCT = randomized controlled trial, DVT = deep venous thrombosis, and PE = pulmonary embolism.

TXA use is routine practice in TKA because of its clinical efficacy and cost-saving benefits. Two retrospective studies analyzed the economic impact of TXA use and reported that TXA administration could produce an estimated cost savings of $879 to $1500 per operation compared with that of placebo^45,46^. Moreover, Irwin *et al*.^10^ reported that a 2-g oral TXA bolus could more effectively reduced transfusion rates and significantly decrease the cumulative hospital cost of around £29,788 in patients undergoing TKA, compared with 15-mg/kg intravenous TXA. In double-blind, randomized, controlled trial comparing oral TXA with IV TXA, Kayupov *et al*. found a significant decrease in pharmacy cost of approximately $33 to $94 per patient in oral TXA group, resulting in a significant cost difference^18^. A randomized controlled trial by Luo *et al*. reported that the application of oral form of TXA could produce a greater pharmacy cost savings, compared with the intravenous and IA formulations in total hip arthroplasty^47^. In our study, the estimated savings of an appropriate oral TXA dose at our center was $13 to $28 for every 60 patients treated compared with an equivalent dose of IA and IV TXA. More than 200,000 TKAs are performed annually in China, and the numbers of this knee surgery will continue to grow over time^48^. Therefore, a switch to oral TXA may yield corresponding cost savings of $2.6 million to $5.6 million annually for our health-care system. Moreover, our randomized controlled trials have indicated that multiple postoperative boluses of oral or IV TXA can effectively reduce inflammatory responses and postoperative blood loss in TKA compared with the single preoperative dose of TXA^20,49^. Therefore, the transition to oral TXA may provide even greater cumulative savings.

This study has several limitations. First, this trial included no placebo group because substantial evidence confirmed the efficacy of TXA at any dose, timing, or route of administration^32,34^ and favored the use of TXA in this setting^10,12^. Withholding of TXA would deprive TKA patients the potential beneficial effects of TXA use, including reduced blood loss, Hb drop, and decreased risk of blood transfusion. Second, no significant differences were identified in terms of transfusions, DVT, and PE. The present study may be underpowered for detecting these meaningful comparisons. However, increasing evidence has confirmed the safety of TXA administration. Third, several potential variations, such as hemodilution, may affect estimated blood loss. However, these variations should cancel each other out because of the randomized design.

In conclusion, this randomized controlled trial indicated that 2 g of oral TXA results in similar blood loss compared to 20 mg/kg of IV or 2 g of IA TXA in TKA with no closed suction drain and tourniquet. The equivalent efficacy, larger cost-saving effects and convenient administration of oral TXA support a transition to oral TXA to reduce health-care costs.

## Data Availability

The datasets generated during and/or analyzed during the current study are available from the corresponding author on reasonable request.
